# Account of a New Poultice

**Published:** 1800-02

**Authors:** T. Payne

**Affiliations:** Surgeon, Brook Street, London


### *Account of a new Poultice, lately proposed by Mr.* T. PAYNE, *Surgeon, Brook Street, London.*

FROM the second edition of a small pamphlet, addressed by Mr. Payne to the Faculty, we extract the following remarks: "It may appear strange," says the Author, "to those who do not enter into the minutiæ of surgery, that I should look upon the proper application of so simple a remedy as that of poultice, as an essential object, (which certainly I do, as well as many other *(deemed)* little things in surgery); but having met with repeated proofs of the necessity of it, I have decidedly formed my opinion on this head. What gave me the first idea respecting the subject of this publication, was, that in the Parish Infirmary of St. George, Hanover-square, to which I have the honour of being surgeon, frequent complaints were made by the patients under my care, that the poultice was uneasy and caused much irritation: this, I supposed, arose from the circumstance of its not being made of bread so good as usual, (an alteration having been made in that article in the house) on which account I thought it my duty to remedy it, if possible, for the ease of the poor afflicted patients. I immediately set about making experiments, many of which I need not mention, as I have formed a poultice which succeeds beyond my most sanguine expectations, and I flatter myself it may prove of some utility. The composition will not only save full half the expence of the former poultice, but the consumption of BREAD CORN entirely; a most desirable object to accomplish in the present scarcity of that article; which affords me infinite pleasure, and I make no doubt will prove a stimulus (if any be necessary) to those gentlemen into whose hands this may fall, to induce them to give it a fair and impartial trial. The particulars and expence attending the late and present poultice in use at the aforesaid Infirmary, per week, are as follow:

"OLD POULTICE.

| | s. | d. |
|---|---|---|
| Bread 49lb. at 2½d. per pound - | 10 | 2½ |
| Milk 14 quarts, at 2d. per quart - | 2 | 4 |
| Lard 2¼lb. at 9d. per pound - - | 1 | 8¼ |
| | 14 | 2¾ |

"NEW

" NEW POULTICE.

| | s. | d. |
|---|---|---|
| Fine Pollard 3½ pecks, at 6d. per peck - | 1 | 9 |
| Genuine Linſeed Flour 14lb. at 4d. per lb. | 4 | 8 |
| Lard, a quarter of pound - - | 0 | 2¼ |
| | 6 | 7¼ |

" From the above ſtatement, I hope, will ſatisfactorily appear, not only the great difference in expence, but the ſaving of forty-nine pounds of bread, per week. Even when oatmeal is uſed for poultices (as is the caſe in ſome hoſpitals) there will be a ſaving of full one-third in the expence; but bread is made of oatmeal in many counties of this kingdom, and by the method above ſtated, that article of ſuſtenance is entirely ſaved. Theſe advantages, are, in my humble opinion, highly worthy the attention of every one who has a fellow feeling for the human ſpecies, and a deſire to be uſeful, in any degree, to his country, in a time of general diſtreſs."

